# Application of PCR-Denaturing-Gradient Gel Electrophoresis (DGGE) Method to Examine Microbial Community Structure in Asparagus Fields with Growth Inhibition due to Continuous Cropping

**DOI:** 10.1264/jsme2.ME11222

**Published:** 2011-12-27

**Authors:** Yasufumi Urashima, Takahiro Sonoda, Yuko Fujita, Atsuko Uragami

**Affiliations:** 1 National Agricultural Research Center, 3–1–1 Kannondai, Tsukuba, Ibaraki, 305–8666 Japan; 2 Government of the Prefecture of Fukushima, 2–16 Sugitsuma-cho, Fukushima City, Fukushima 960–8670, Japan; 3 Aizu Agriculture and Forestry Office, Fukushima Prefecture Government, 7–5 Outemachi, Aizuwakamatu City, Fukushima 965–8501, Japan; 4 National Institute of Vegetable and Tea Science, 3–1–1 Kannondai, Tsukuba, Ibaraki 305–8666, Japan

**Keywords:** asparagus, PCR-DGGE, *Fusarium oxysporum*, *Fusarium proliferatum*, continuous cropping

## Abstract

Growth inhibition due to continuous cropping of asparagus is a major problem; the yield of asparagus in replanted fields is low compared to that in new fields, and missing plants occur among young seedlings. Although soil-borne disease and allelochemicals are considered to be involved in this effect, this is still controversial. We aimed to develop a technique for the biological field diagnosis of growth inhibition due to continuous cropping. Therefore, in this study, fungal community structure and *Fusarium* community structure in continuously cropped fields of asparagus were analyzed by polymerase chain reaction/denaturing-gradient gel electrophoresis (PCR-DGGE). Soil samples were collected from the Aizu region of Fukushima Prefecture, Japan. Soil samples were taken from both continuously cropped fields of asparagus with growth inhibition and healthy neighboring fields of asparagus. The soil samples were collected from the fields of 5 sets in 2008 and 4 sets in 2009. We were able to distinguish between pathogenic and non-pathogenic *Fusarium* by using Alfie1 and Alfie2GC as the second PCR primers and PCR-DGGE. Fungal community structure was not greatly involved in the growth inhibition of asparagus due to continuous cropping. By contrast, the band ratios of *Fusarium oxysporum* f. sp. *asparagi* in growth-inhibited fields were higher than those in neighboring healthy fields. In addition, there was a positive correlation between the band ratios of *Fusarium oxysporum* f. sp. *asparagi* and the ratios of missing asparagus plants. We showed the potential of biological field diagnosis of growth inhibition due to continuous cropping of asparagus using PCR-DGGE.

Asparagus (*Asparagus officinalis* L.) is a long-term perennial vegetable crop. A certain number of years after planting, yield and quality start to decline due to natural aging. In addition, when marketable yields continually decrease as a result of growth suppression, it becomes necessary to replant; however, asparagus production in former asparagus fields (replanting) is often less profitable than that in fresh fields without a history of asparagus crops (new planting). This phenomenon is common in older asparagus production areas and is known as the replant problem (1, 7, 16, 37). Growth inhibition due to continuous cropping of asparagus has become a large problem in the world’s older asparagus production areas.

A number of factors have been associated with the early decline of asparagus, including increased infection of the soil with soil-borne pathogenic fungi, mainly *Fusarium oxysporum* f. sp. *asparagi* and *Fusarium proliferatum*(1, 26, 34). *F. oxysporum* and *F. proliferatum* are the casual agents of a destructive disease of asparagus called *Fusarium* crown and root rot. Both species are ubiquitous in asparagus field soils and colonize the crowns and roots of symptomatic, as well as asymptomatic, plants (33). *F. oxysporum* is more often found in young roots, whereas *F. proliferatum* is dominant in nearly all other plant parts (4). These pathogens can be transmitted by seeds, and can simultaneously colonize both vascular and epidermal tissues.

Furthermore, growth inhibition due to allelochemicals from the asparagus residue and to the degradation of field environments, such as the physical condition and chemical properties of the soil, seem to be incidental factors in the early decline of asparagus (6, 21, 36). So far, effective countermeasures have not been established, and technological development in this area is expected.

Many studies have focused on the effects of soil-borne fungus on asparagus (1, 3, 4, 19, 26); however, little research has focused on the effects of soil microorganism community structures on asparagus. Soil microorganisms directly and indirectly affect crop growth, whereas fertilization, organic substance management, and cropping systems affect the soil microorganism community structure. Recently, it has become possible to extract DNA directly from the soil (environmental DNA), and a method has been developed for estimating the soil microorganism community structure from the environmental DNA. Muyzer *et al.* was the first to profile microbial communities using polymerase chain reaction and denaturing-gradient gel electrophoresis (PCR-DGGE) (23). The first application of this technique for fungal community analysis was performed by Kowalchuk *et al.*(15). Since then, PCR-DGGE has proven to be a powerful technique for the culture-independent detection and characterization of fungal populations in plant materials and soil. Yergeau *et al.* used PCR-DGGE to profile *Fusarium* community composition in asparagus plant samples (35). The PCR-DGGE technique could be used to assess *Fusarium* community composition directly from plant samples, without the need for isolation and culture; however, no study has tried to assess *Fusarium* community composition in soil.

In this study, we examined fungal and *Fusarium* community structures in asparagus fields and compared these structures in continuously cropped fields of asparagus with growth inhibition and healthy neighboring fields of asparagus. We judged that microbial diagnosis could determine fields with a high possibility of early decline by examining the *Fusarium* community structure. The last goal of this research was to develop a microbial diagnosis method for asparagus fields experiencing growth inhibition due to continuous cropping.

## Materials and Methods

### Sampling site

Soil samples were collected in August 2008 and 2009 in the Aizu region of Fukushima Prefecture, Japan; samples were taken from both continuously cropped fields of asparagus with growth inhibition and healthy neighboring fields of asparagus. Soil analysis was carried out as a set of fields of asparagus with growth inhibition and healthy neighboring fields of asparagus. Soil samples were collected from the fields as 5 sets in 2008 and 4 sets in 2009. All soil samples were taken at a depth of 15–25 cm and collected from the position which left 5 cm of the asparagus plant. The soil was collected from 5 places in one field.

Soil samples were passed through a 2 mm sieve to remove plant debris, and the microbial biomass was immediately analyzed. Microbialbiomasscarbonwasmeasuredbyachloroform-fumigation-extraction method (32). For DNA extraction, the soil samples were stored at −20°C. For soil chemical analysis, the soil samples were air dried. The number of missing plants in the field was divided by all the plants, and the ratio of missing asparagus plants was calculated. The number of missing plants in the field was investigated in October 2009.

### Analysis of soil properties

Soil pH and electrical conductivity (EC) were determined in a 1:5 suspension of each soil sample. Available phosphate was determined by Truog P (30). Total carbon and nitrogen were measured using Vario MAX (Elementar, Hanau, Germany). Exchangeable cations (12) were measured using ICP-Atomic Emission Spectrometry (ICP-AES, Vista-MPX; Seiko Instruments, Chiba, Japan).

### DNA extraction

DNA was extracted according to the method of Hoshino and Matsumoto (7). DNA was extracted from 400 mg fresh soil using a FastDNA Spin kit for soil (Q-Biogene/MP Biomedicals, Solon, USA) according to the manufacturer’s recommendations, except that the DNA was eluted in 80 μL DES (DNA Elution Solution) in the final step (20). Skim milk (20%, 80 μL) was added to the extraction buffer to avoid DNA adsorption to clay particles in some types of Andosol (7).

### PCR amplification of fungal 18S rDNA fragment

PCR amplification of 18S rRNA genes was performed with a primer set for fungus (NS1, GCFung) (10, 11, 16). The 50 μL PCR mixture contained 5 μL of 10×PCR buffer, 5 μL of each primer (10 μM), 1 μL of 10 mM dNTP mix, and 1.25 U of KOD-plus DNA polymerase (Toyobo, Osaka, Japan). KOD-plus has shown higher PCR efficacy than other DNA polymerases, especially in soil DNA containing humic acid (10). The PCR program consisted of initial denaturation at 94°C for 2 min, followed by 28 cycles of denaturation at 94°C for 15 s, annealing at 50°C for 30 s, and extension at 68°C for 30 s. PCR was conducted with a GeneAmp PCR System 9700 (Applied Biosystems/Life Technologies, Carlsbad, USA). The products were quantified on a 1.5% agarose gel using *Hind*III-digested λ DNA as the standard.

### *Fusarium* strains

*F. oxysporum* AF847, *F. oxysporum* AF3823, *F. proliferatum* AF860 and *F. proliferatum* AF5822 were isolated from asparagus fields in Nagano Prefecture, Japan. *F. oxysporum* f. sp. *asparagi* were isolated from asparagus fields in Hokkaido Prefecture, Japan. *F. oxysporum* f. sp. *raphani*, *F. oxysporum* f. sp. *cucumerinum*, *F. oxysporum* f. sp. *spinaciae* were provided by Ikuo Kadota (National Agricultural Research Center for the Tohoku Region [NARCT]).

### PCR amplification of *Fusarium sp.*

A nested PCR procedure was used to amplify elongation factor-1α (EF-1α) from *Fusarium* DNA extracted from soil samples or *Fusarium* strains. The first PCR step was performed using EF-1 (5′-ATGGGTAAGGARGACAAGAC-3′) and EF-2 (5′-GGARGTACC AGTSATCATGTT-3′) primers (25). The amplicons were subsequently diluted and reamplified using Alifie1-GC (5′-CGCCCGC CGCGCGCGGCGGGCGGGGCGGGGGCACGCGGGGTCGTC ATCGGCCACGTCGACTC-3′) and Alfie2 (5′-CCTTACCGAGC TCRGCGGCTT-3′) primers, as described by Yergeau *et al.*(35), or Alfie1 (5′-TCGTCATCGGCCACGTCGACTC-3′) and Alfie2-GC (5′-CGCCCGCCGCGCGCGGCGGGCGGGGCGGGGGCAC GCGGGGCCTTACCGAGCTCRGCGGCTT-3′) primers. All reactions were carried out in 50 μL volumes containing 5 μL of 10×PCR buffer, 5 μL of each primer (10 μM), 1 μL of 10 mM dNTP mix, and 1.25 U of KOD-plus (Toyobo, Osaka, Japan). PCR was conducted with a GeneAmp PCR System 9700 (Applied Biosystems/Life Technologies).

### DGGE analysis

DGGE analysis was performed using the DCode System (Bio-Rad Laboratories, Hercules, CA). In the case of fungi, gel electrophoresis was carried out for 18 h at 50V on a 7% acrylamide/bis-acrylamide (37.5:1) gel with a 20–45% denaturant gradient (100% denaturant corresponding to 7 M urea and 40% [v/v] formamide) (16, 20).

In the case of *Fusarium*, gel electrophoresis was carried out for 17 h at 110V on a 7% acrylamide/bis-acrylamide (37.5:1) gel with a 40–60% denaturant gradient. The improved PCR method of Yergeau *et al.*(35) was used; namely, the annealing temperature of the first PCR was changed from 50°C to 60°C. In addition, the primer that attached to the GC clamp was changed to the Alfie2. Gels were stained with SYBR Green I for 30 min, photographed with a ChemDoc XRS (Bio-Rad Laboratories), and analyzed with Quantity One Software and FingerPrinting II software (Bio-Rad Laboratories).

### Statistical analysis

All data were analyzed by ANOVA and, where a significant effect was observed, Tukey’s HSD test was applied for comparison of treatment means using STATISTICA 06J software (StatSoft, Tokyo, Japan). Dendrograms were generated based on the unweighted pair group with the arithmetic mean (UPGMA) clustering method.

## Results and Discussion

### Analysis of soil properties

Table 1 shows the soil chemical properties and biomass carbon values of the investigated fields. The pH and EC values in most fields were optimal or slightly lower pH, higher EC based on standard value for soil diagnostics (pH 6.0–6.5, EC <0.3 dS m^−1^ (time of fertilizer application), the standard application rate of fertilizer in Fukushima Prefecture). In contrast, the available phosphoric acid, exchangeable potassium and calcium in many fields were high based on the standard value for soil diagnostics (available phosphoric acid <0.2 g kg^−1^ soil, exchangeable potassium = 0.15–0.3 g kg^−1^ soil, calcium = 2–3 g kg^−1^ soil). In some fields, the available phosphoric acid exceeded 1 g kg^−1^. Murakami *et al.* reported that the excess application of phosphate to soils promotes the incidence of clubroot disease (22). The relationship between excess phosphate in the soil and the incidence of asparagus diseases is a subject for future study.

In the Aizu region of Fukushima Prefecture, many asparagus farmers use cattle feces as compost. On average, these farmers apply cattle feces compost at levels up to 20 t ha^−1^ every other year. Furthermore, some exemplary farmers use up to 20 t ha^−1^ of cattle feces compost every year. The application of this compost has been reported to raise the available phosphoric acid and exchangeable potassium levels (14); therefore, the levels of available phosphoric acid and exchangeable potassium were high in many of the investigation fields. In addition, the exchangeable calcium content was high because lime is used for pH improvement. The pH, Biomass-C, exchangeable potassium and calcium levels often showed a high value in continuously cropped asparagus fields with growth inhibition; however, the difference was not marked and was not thought to inhibit growth. As an explanation of the high values in growth inhibited fields, it is supposed that cultivation had been repeated for many years; however, we were not able to identify any characteristic chemical properties of continuously cropped asparagus fields with growth inhibition. Similarly, there was no relationship between growth inhibition due to continuous cropping and microbial biomass carbon values. Because the investigated fields were adjacent, soil physical properties and drainage conditions were likely to have been similar among fields. Thus, we hypothesized that the chemical and physical properties of the soil have little effect on the poor growth of asparagus and that the microbial community structure has an influence on the growth of asparagus in this region.

### PCR-DGGE analysis of fungal 18S rDNA fragment

**Fig. 1:**
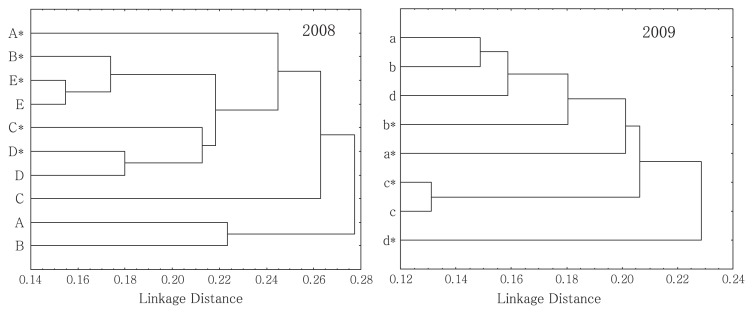
Cluster analysis of 18S rDNA denaturing gradient gel electrophoresis profiles of asparagus fields. The same letter shows asparagus fields of the same farmers. Asterisk indicates growth injury due to continuous asparagus cropping in a field. The dendrogram was constructed using the unweighted pair group method with arithmetic mean analysis (UPGMA). Diversity index (Shannon-Wiener Diversity Index, *H’*=−∑Pi(lnPi))): A (2.47), A* (2.96), B (2.49), B* (2.81), C (2.62), C* (2.77), D (2.80), D* (2.87), E (2.96), E* (2.66), a (2.68), a* (2.93), b (2.75), b* (2.66), c (2.65), c* (2.73), d (3.12), d* (3.12).

There was no significant difference in the DGGE banding pattern of the 18S rRNA gene between continuously cropped fields of asparagus with growth inhibition and good growth fields. The results were subjected to cluster analysis and visualized on a dendrogram (Fig. 1). In both years, the cluster was not clearly divided between continuously cropped fields with growth inhibition and good growth fields. As a result, we surmised that the fungal community structure was not greatly involved in growth inhibition in continuously cropped fields. Also, the Shannon diversity indexes of the fungal community structures showed no consistent trend in either year, and the Shannon diversity indexes in continuously cropped fields of asparagus were not low (data not shown).

Some studies have reported that the soil microbial community structure was affected by plant nutrient management techniques, such as cropping systems, tillage systems, fertilization, and fumigation. Suzuki *et al.* reported that fungal communities were related to fertilization methods such as manure application and concluded that fungal communities might be sensitive to soil environmental changes (29). In addition, Sekiguchi *et al.* reported that green manure applications changed the fungal DGGE profile (27). Fertilization techniques such as compost application changed the fungal community structure greatly. In this study, most of the investigated fields—including both continuously cropped fields and good growth fields—had been fertilized with cattle feces compost. It is clear that compost application affected the fungal community structures, and so we concluded that fungal community structures might not differ between continuously cropped fields and good growth fields of asparagus. We would like to focus our analysis on the community structure of *Fusarium*, including pathogenic fungi, because there was no a marked difference in fungal community structure.

### Discrimination of pathogenicity and nonpathogenic *Fusarium sp.* by PCR-DGGE

**Fig. 2:**
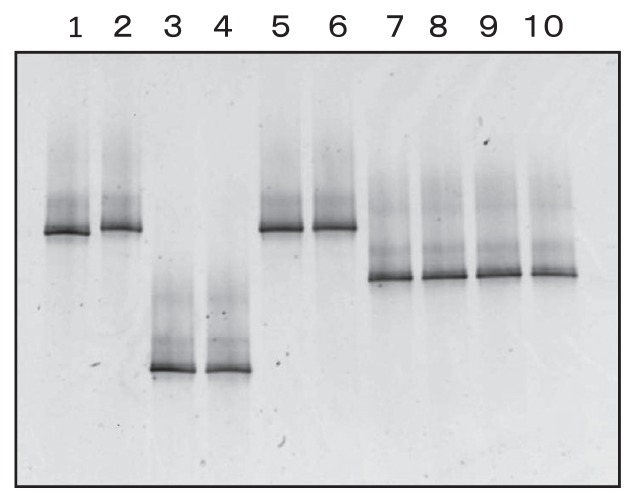
DGGE band pattern of *Fusarium* sp.Lanes 1–6 show pathogenic fungi of asparagus. Lane 1: *F. oxysporum* AF847; Lane 2: *F. oxysporum* AF3823; Lane 3: *F. proliferatum* AF860; Lane 4: *F. proliferatum* AF5822; Lane 5: *F. oxysporum* f. sp. *asparagi* 1; Lane 6: *F. oxysporum* f. sp. *asparagi* 2; Lane 7: *F. oxysporum* f. sp. *raphani*; Lane 8: *F. oxysporum* f. sp. *cucumerinum*; Lane 9: *F. oxysporum* f. sp. *spinaciae* 1; Lane 10: *F. oxysporum* f. sp. *spinaciae*.

We extracted DNA from *Fusarium* sp., which cause disease in asparagus and other crops, and PCR-DGGE was carried out based on the method of Yergeau *et al.*(35); however, there were multiple bands from the *Fusarium* sp. isolates. Yergeau *et al.* also reported multiple banding patterns from *F. oxysporum* f. sp. *dianthi* and *F. proliferatum* isolates. For this reason, improvement of the method was necessary for DGGE analysis of *Fusarium*. First, the annealing temperature of the first PCR was changed from 50°C to 60°C. In addition, the primer that attached to the GC clamp was changed to Alfie2. The GC clamp alters the melting properties of the fragment; this change greatly increased the detectable fractions. Using appropriate conditions, the attachment of a GC clamp can increase the detection of single base-pair change to nearly 100% (24, 28). As a result, it was confirmed that the DGGE result of isolated *Fusarium* was a single band (Fig. 2). The band positions differed between *Fusarium* sp. that caused disease in asparagus and other crops. Thus, we could analyze disease-causing *F. oxysporum* and other *F. oxysporum* separately with this method.

### PCR-DGGE analysis of *Fusarium sp.* in asparagus field soils

**Fig. 3:**
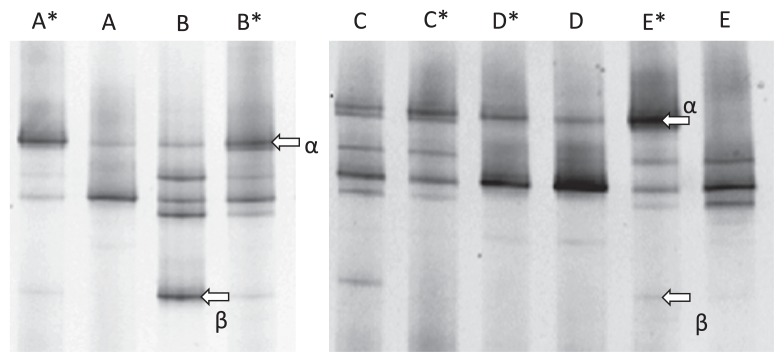
DGGE profiles of *Fusarium* sp. community in an asparagus field. Arrow α: *F. oxysporum* f. sp. *asparagi*. Arrow β: *F. proliferatum*. A given asparagus field is represented by the same letter. Asterisk indicates growth injury due to continuous asparagus cropping field. This gel pattern shows results from 2008.

**Fig. 4:**
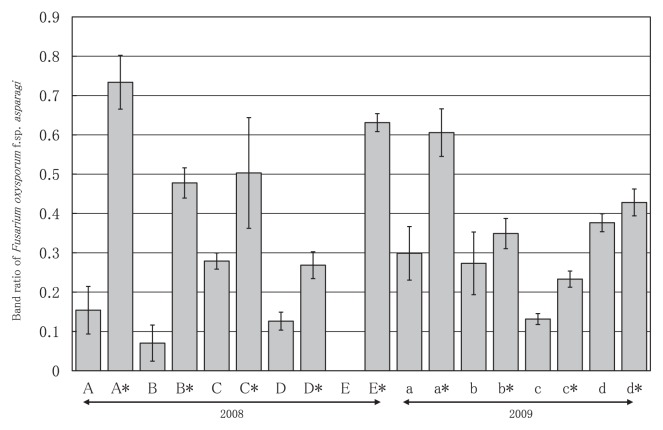
Band ratio of *Fusarium oxysporum* f. sp. *asparagi* in an asparagus field. A given asparagus field is represented by the same letter. Asterisk indicates growth injury due to continuous asparagus cropping in a field. Error bars represent the standard deviation (n=3). There were significant differences among field sets at *p*<0.05 (Tukey’s HSD test) except for field b and b* in 2009.

The DGGE profiles of the *Fusarium* in the investigated fields are shown in Fig. 3. In the case of continuously cropped fields with growth inhibition, there was a thick band (indicated by arrow α) in the upper part of the lanes. This band was identified as *F. oxysporum* f. sp. *asparagi*. By contrast, there was no thick band in the upper part of the lanes in samples from healthy neighboring fields of asparagus. Thus, we surmised that the growth of asparagus was reduced when the intensity of this band was high and proceeded to examine the band intensity ratios of the investigated fields (Fig. 4). The band intensity ratio of a field with growth inhibition was higher than that of the corresponding healthy neighboring field.

The band in the lower part of the lanes (indicated by arrow β) was *F. proliferatum*, which is pathogenic to asparagus (Fig. 3). There was no consistent trend between the occurrence of the growth inhibition of asparagus and the band intensity ratio in the lower part of the lanes. Moreover, this band was not detected in some fields. For these reasons, we concluded that soil disease in asparagus fields in the Aizu region of Fukushima Prefecture was mainly caused by *F. oxysporum* f. sp. *asparagi. F. proliferatum* has been a frequent isolate in the warmer climates of southern Italy (19), North and Central America, Australia, and South Africa (3). In contrast, Blok and Bollen failed to recover any *F. proliferatum* isolates in the Netherlands (1) due to the cooler soil temperature; therefore, because Fukushima Prefecture is located in a cold region of Japan, it seems reasonable to conclude that soil disease is mainly caused by *F. oxysporum* f. sp. *asparagi*.

**Fig. 5:**
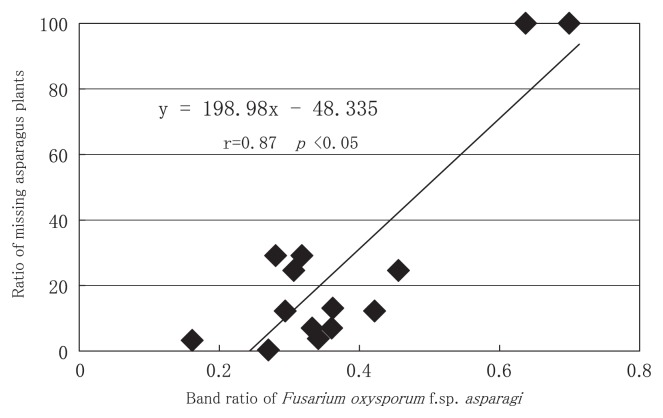
Correlation between the band ratio of *Fusarium oxysporum* f. sp. *asparagi* and the ratio of missing asparagus plants. These data shows results from 2009.

Because the correlation between the growth of asparagus and *F. oxysporum* f. sp. *asparagi* band intensity was expected, we calculated the correlation. There was a positive correlation (r=0.93, p<0.05) between the band ratio of *F. oxysporum* f. sp. *asparagi* and the ratio of missing plants in an asparagus field (Fig. 5). These results led us to conclude that the PCR-DGGE method is effective for field diagnosis because we can estimate the ratio of missing plants in a field of asparagus from the band ratio of *F. oxysporum* f. sp. *asparagi*. Although a significant correlation was found between the band ratio of *F. oxysporum* f. sp. *asparagi* and the ratio of missing asparagus plants, further investigations will be necessary because the number of investigated fields in this study was not sufficient.

## Conclusions

The PCR-DGGE method could take on a substantial role in the diagnosis of asparagus fields, and may eventually enable field diagnosis similar to a human health examination. PCR-DGGE is an efficient method that can detect *Fusarium* directly from the soil without an isolation step. Previous methods required prior cultivation of *Fusarium* isolates, which limited the number of possible analyses. The PCR-DGGE method is easy and rapid and makes it possible to investigate many fields in a short time.

Here, we showed the potential of the field diagnosis of growth inhibition due to continuous cropping *via* PCR-DGGE analysis of the *Fusarium* community structure. With this method, growth inhibition in a continuously cropped field may be avoided by carrying out a field diagnosis before replanting asparagus. In cases where the intensity of the band representing *F. oxysporum* f. sp. *asparagi* is low, the field can be judged as healthy, and the planting of asparagus seedlings in that field can be recommended. In contrast, when the band intensity of *F. oxysporum* f. sp. *asparagi* is high, the field can be judged as growth inhibited. Soil disinfection is required in such a field. There are many methods for soil disinfection, including chloropicrin fumigation, soil solarization (13), steam sterilization (31), and soil reduction (18). For reasons of environmental conservation and the health of farmers, it is preferable to avoid methods using agricultural chemicals and, instead, to perform soil disinfestation and remediation with microbes (2, 5).

In conclusion, PCR-DGGE analysis of the *Fusarium* community structure could be used for the biological field diagnosis of asparagus, allowing the assessment of *Fusarium* community structure directly from the soil, without the need for isolation and culture.

## Figures and Tables

**Table 1:** Chemical and microbial properties of sampled fields

Field^a^		pH	EC (dS m^−1^)	Truog-P (g kg^−1^ soil)	Exchangeabel cation (g kg^−1^ soil)			Total C (g kg^−1^ soil)	Total N (g kg^−1^ soil)	Biomass C (g kg^−1^ soil)

					K_2_O	CaO	MgO			
2008	A	5.96	0.227	0.27	0.66	3.08	0.57	34.3	3.21	1.03
	A*	6.14	0.226	1.69	1.06	5.60	0.69	43.7	3.26	1.24
	B	5.17	0.315	0.50	0.50	1.10	0.25	27.6	2.37	0.70
	B*	5.33	0.614	0.50	0.81	1.95	0.44	20.8	2.05	0.73
	C	5.97	0.470	0.93	0.58	2.83	0.46	19.4	1.63	0.76
	C*	5.94	0.223	0.88	0.54	2.59	0.43	17.8	1.81	0.85
	D	5.31	0.742	0.81	1.22	5.32	0.63	49.0	3.80	0.78
	D*	5.69	0.488	0.62	1.03	6.60	0.72	51.9	3.84	1.13
	E	4.84	0.164	0.24	0.46	1.70	0.30	40.9	2.91	0.97
	E*	6.03	0.239	1.65	1.20	4.80	1.58	45.0	3.42	1.03

2009	a	6.12	0.228	0.27	0.93	2.51	0.51	30.7	2.69	1.33
	a*	6.40	0.178	1.82	1.02	5.65	0.76	41.0	3.00	1.49
	b	5.06	0.420	0.60	0.63	1.06	0.19	30.5	2.40	0.95
	b*	5.46	0.309	0.51	0.67	1.59	0.25	20.6	1.80	0.85
	c	5.82	0.213	0.29	0.34	2.04	0.48	21.1	1.74	0.97
	c*	5.94	0.177	0.36	0.48	2.35	0.65	20.2	1.75	1.15
	d	5.88	0.224	0.94	0.45	3.19	0.35	44.8	3.07	0.79
	d*	5.99	0.147	0.48	0.43	2.66	0.44	18.3	1.49	0.73

Each value is the mean (n=3).

a A given asparagus field is represented by the same letter. Upper case, samples from 2008; lower case, samples from 2009. Asterisk indicates growth injury due to continuous asparagus cropping in a field.

## References

[1] Blok WJ, Bollen GJ (1995). Fungi on roots and stem bases of asparagus in the Netherlands: species and pathogenicity. Eur J Plant Pathol.

[2] Blok WJ, Lamers JG, Termorshuizen AJ, Bollen GJ (2000). Control of soilborne plant pathogens by incorporating fresh organic amendments followed by tarping. Phytopathology.

[3] Elmer WH (2000). Incidence of infection of asparagus spears marketed in Connecticut by *Fusarium* spp. Plant Dis.

[4] Gilbertson RL, Manning WJ (1983). Contamination of asparagus flowers and fruit by airborne spores of *Fusarium moniliforme*. Plant Dis.

[5] Goud JKC, Termorshuizen AJ, Blok WJ, van Bruggen AHC (2004). Long-term effect of biological soil disinfestation on *Verticillium* wilt. Plant Dis.

[6] Hartung AC, Nair MG, Putnam AR (1990). Isolation and characterization of phytotoxic compounds from asparagus (*Asparagus officinalis* L.) roots. J Chem Ecol.

[7] Hartung AC, Stephenes CT (1983). Effect of allelopathic substances produced by asparagus on incidence and severity of asparagus decline due to *Fusarium* crown rot. J Chem Ecol.

[8] Hoshino YT, Matsumoto N (2004). An improved DNA extraction method using skim milk from soils that strongly adsorb DNA. Microbes Environ.

[9] Hoshino YT, Matsumoto N (2007). Change in fungal community structure in bulk soil and spinach rhizosphere soil after chemical fumigation as revealed by 18S rDNA PCR-DGGE. Soil Sci Plant Nutr.

[10] Hoshino YT, Matsumoto N (2008). Comparison of 18S rDNA primers for estimating fungal diversity in agricultural soils using polymerase chain reaction-denaturing gradient gel electrophoresis. Soil Sci Plant Nutr.

[11] Hoshino YT, Morimoto S (2010). Soil clone library analyses to evaluate specificity and selectivity of PCR primers targeting fungal 18S rDNA for denaturing-gradient gel electrophoresis (DGGE). Microbes Environ.

[12] Kamewada K, Shibata K (1997). Simple extraction method for measuring exchangeable cations in soils that is not required measuring cation exchange capacity (in Japanese). Jpn Soc Soil Sci Plant Nutr.

[13] Katan J, Greenberger A, Alon H, Grinstein A (1976). Solar heating by polyethylene mulching for the control of diseases caused by soilborne pathogens. Phytopathology.

[14] Katano Y, Kinoshita T, Takei A (1998). The effect of successive organic matter applications on soil chemical properties in a greenhouse (in Japanese). Res. Bull. Aichi-ken Agric Ctr.

[15] Kowalchuk GA, Gerards S, Woldendrop JW (1997). Detection and characterization of fungal infections of *Ammophila arenaria*(marram grass) roots by denaturing gradient gel electrophoresis of specifically amplified 18S rDNA. Appl Environ Microbiol.

[16] Lake RJ, Falloon PG, Cook DWM (1993). Replant problem and chemical components of asparagus roots. New Zeal J Crop Hort Sci.

[17] May LA, Smiley B, Schmidt MG (2001). Comparative denaturing gradient gel electrophoresis analysis of fungal communities associated with whole plant corn silage. Can J Microbiol.

[18] Momma N (2008). Biological soil disinfection (BSD) of soilborne pathogens and its possible mechanisms. Japan Agr. Res Quartely.

[19] Moretti A, Logrieco A, Doko B, Frisullo S, Visconti A, Bottalico A (1997). *Fusarium proliferatum* from asparagus in Italy: Occurrence, fertility and toxigenicity. Cereal Res Commun.

[20] Morimoto S, Hoshino YT (2008). Methods for analysis of soil communities by PCR-DGGE (1): Bacterial and fungal communities (in Japanese). Soil Microorganisms.

[21] Motoki S, Nishimura E, Kiyazawa H, Hiradate S, Shinohara Y (2006). Participation of allelopathy in injury due to continuous cropping of asparagus (*Asparagus officinalis* L.) in alluvial soil (in Japanese). Hort. Res Japan.

[22] Murakami K, Nakamura F, Goto I (2004). The causal relationship between excess phosphate in the soil and incidence for clubroot disease (in Japanese). Jpn J Soil Sci Plant Nutr.

[23] Muyzer G, de Waal EC, Uitterlinden AG (1993). Profiling of complex microbial populations by denaturing gradient gel electrophoresis analysis of polymerase chain reaction-amplified genes coding for 16S rRNA. Appl Environ Microbiol.

[24] Myers RM, Fischer SG, Lerman LS, Maniatis T (1985). Nearly all single base substitutions in DNA fragments joined to a GC-clamp can be detected by denaturing gradient gel electrophoresis. Nucleic Acids Res.

[25] O’Donnell K, Kistler HC, Cigelnik E, Ploetz RC (1998). Multiple evolutionary origins of the fungus causing Panama disease of banana: Concordant evidence from nuclear and mitochondrial gene genealogies. Proc. Natl. Acad. Sci USA.

[26] Schreuder W, Lamprecht SC, Marasas WFO, Calitz FJ (1995). Pathogenicity of three *Fusarium* species associated with asparagus decline in South Africa. Plant Dis.

[27] Sekiguchi H, Kushida A, Takenaka S (2007). Effect of cattle manure and green manure on the microbial community structure in upland soil determined by denaturing gradient gel electrophoresis. Microbes Environ.

[28] Sheffield VC, Cox DR, Lerman LS, Myers RM (1989). Attachment of a 40-base-pair G+C-rich sequence (GC-clamp) to genomic DNA fragments by the polymerase chain reaction results in improved detection of single-base changes. Proc. Natl. Acad. Sci USA.

[29] Suzuki C, Nagaoka K, Shimada A, Takenaka M (2009). Bacterial communities are more dependent on soil type than fertilizer type, but the reverse is true for fungal communities. Soil Sci Plant Nutr.

[30] Truog E (1930). The determination of the readily available phosphorus of soils. J Amer Soc Agron.

[31] Uematsu S, Nishi K, Kita N (2003). Hot water soil sterilization begins in Japan. Farming Japan.

[32] Vance ED, Brookes PC, Jenkinson DS (1987). An extraction method for measuring soil microbial biomass C. Soil Biol Biochem.

[33] Vujanovic V, Hamel C, Jabaji-Hare S, St-Arnaud M (2002). Development of a selective myclobutanil agar (MBA) medium for the isolation of *Fusarium* species from asparagus fields. Can J Microbiol.

[34] Vujanovic V, Hamel C, Yergeau E, St-Arnaud M (2006). Biodiversity and biogeography of *Fusarium* species from northeastern North American asparagus fields based on microbiological and molecular approaches. Microb Ecol.

[35] Yergeau E, Filion M, Vujanovic V, St-Arnaud M (2005). A PCR-denaturing gradient gel electrophoresis approach to assess *Fusarium* diversity in asparagus. J. Microbiol Methods.

[36] Young CC (1984). Autointoxication in root exudates of *Asparagus officimalis* L. Plant Soil.

[37] Young CC, Chou TC (1985). Autointoxication in residues of *Asparagus officinalis* L. Plant Soil.

